# Short Course High Dose Radiotherapy in the Treatment of Anaplastic Thyroid Carcinoma

**DOI:** 10.1155/2014/764281

**Published:** 2014-10-14

**Authors:** Mark J. Stavas, Eric T. Shinohara, Albert Attia, Matthew S. Ning, Jeffrey M. Friedman, Anthony J. Cmelak

**Affiliations:** ^1^Department of Radiation Oncology, Vanderbilt University Medical Center, Nashville, TN 37232, USA; ^2^Department of Radiation Oncology, Vanderbilt University School of Medicine, 1301 Medical Center Drive, B913/TVC, Nashville, TN 37232, USA

## Abstract

*Purpose*. Anaplastic thyroid carcinoma (ATC) is a rare but aggressive tumor with limited survival. To date, the ideal radiation treatment schedule, one that balances limited survival with treatment efficacy, remains undefined. In this retrospective series we investigate the effectiveness and tolerability of hypofractionated radiation therapy in the treatment of ATC.* Methods*. 17 patients with biopsy proven ATC treated between 2004 and 2012 were reviewed for outcomes and toxicity. All patients received short course radiation.* Results*. The most commonly prescribed dose was 54 Gy in 18 fractions. Median survival was 9.3 months. 47% of patients were metastatic at diagnosis and the majority of patients (88%) went on to develop metastasis. Death from local progression was seen in 3 patients (18%), 41% experienced grade 3 toxicity, and there were no grade 4 toxicities.* Conclusions*. Here we demonstrated the safety and feasibility of hypofractionated radiotherapy in the treatment of ATC. This approach offers shorter treatment courses (3-4 weeks) compared to traditional fractionation schedules (6-7 weeks), comparable toxicity, local control, and the ability to transition to palliative care sooner. Local control was dependent on the degree of surgical debulking, even in the metastatic setting.

## 1. Introduction

Anaplastic thyroid carcinoma (ATC) is a rare but deadly tumor with a median survival of 5-6 months and less than 20% survival at one year [1, 2]. ATC occurs with a slight female predominance (1.5 : 1 ratio) with a peak incidence in the sixth and seventh decades of life [3]. The majority of patients (>75%) develop distant metastasis either at the time of diagnosis or shortly thereafter, with the lungs being the most commonly affected organ [4]. Despite its low incidence, ATC accounts for up to 39% of thyroid cancer deaths [2] with disease-specific mortality approaching 100% [5].

In addition to its poor prognosis, ATC causes significant site-specific morbidity. Commonly, individuals present with a rapidly enlarging neck mass causing symptoms of dysphagia, odynophagia, dyspnea, anxiety, and vocal cord paralysis. Unless aggressive treatments are applied, such as surgical resection and external beam radiation therapy (EBRT), patients die from uncontrolled local progression causing suffocation and massive bleeding [6, 7]. Furthermore, rapid airway obstruction remains a major cause of death even in patients undergoing tracheostomy [8, 9]. Therefore, treatment approaches should emphasize the importance of local control even in the metastatic setting.

Given the morbidity and poor prognosis of ATC, palliative and supportive care remain an essential part in the management of these patients [5, 10]. Owing to disappointing results with current treatments strategies, there have been many attempts to improve clinical outcomes. The most promising one, in terms of local control, has been with trimodality therapy combining surgery with chemotherapy and radiation [11]. Various altered fractionation schedules have been proposed such as twice daily treatments or protracted once daily courses, but these schedules do not consider the challenges posed upon patients with limited survival. Two of the main concerns with twice daily treatments are the burden of transportation and association with significant acute toxicities. Furthermore, even with the use of standard fraction sizes and traditional 30-day treatment courses, treatment related side effects tend to be serious, requiring hospitalizations, parenteral nutrition, and delays in supportive care [12].

To date, an optimal radiation approach to ATC (one that considers both outcomes and quality of life) has not been defined. Because of the significant toxicities and logistical challenges related to prolonged or twice daily treatment schedules, our institution has shifted to treat ATC with maximum surgical debulking followed by short course high dose (hypofractionated) radiotherapy with or without chemotherapy. From our experience, shorter treatment courses minimize the challenges associated with transportation and on-treatment toxicity. In addition, shorter courses may lessen the burden of care in this population, especially when the concern for long-term radiation effects is less critical. Most importantly, the quality of life in patients with limited survival should be of highest priority. Here we retrospectively review our use of hypofractionated radiotherapy in the treatment of ATC.

## 2. Methods

### 2.1. Patients

Following institutional review board approval, patients with pathologically proven ATC referred to the department of radiation oncology between 2004 and 2012 were retrospectively reviewed for outcomes and treatment toxicity. All patients received ≥2.5 Gy dose per fraction (median dose 3 Gy (range 2.5–4 Gy)). Patients with metastases at the time of diagnosis were included because local control of the primary site was a major issue in this population.

### 2.2. Treatment

**Figure 1:**
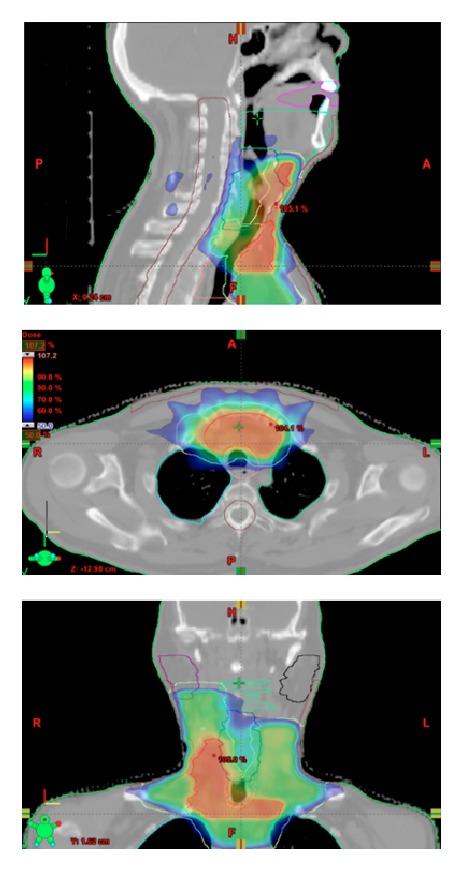
Radiation dose distribution for a 65-year-old male with ATC, status post gross total resection with involved cervical nodes. 51 Gy delivered in 17 fractions.

All patients underwent a computed tomography simulation for treatment planning. They were placed in a long thermoplastic mask for immobilization with their neck in maximum extension. The treatment volumes were constructed using presurgical cranial/caudal margins and the postsurgical axial margins including the entire thyroid bed. Typical fields for the primary tumor extended from just below the hyoid bone to the level VI cervical and upper mediastinal lymph nodes (Figure 1). Only the involved and at risk nodal levels were treated. Megavoltage external beam radiation was delivered using intensity modulated radiation therapy (IMRT) and Eclipse planning software. Avoidance structures such as the trachea, esophagus, and spinal cord were contoured and well-defined dose constraints were applied. The maximum spinal cord dose was ≤36 Gy in 3 Gy fractions. Patients were treated once daily Monday through Friday. Treatment toxicity and follow-up images were reviewed in the medical record.

### 2.3. Follow-Up and Endpoints

Patients were assessed during treatment and subsequently every 2–4 weeks after treatment for both response and toxicity. Local control and treatment response were retrospectively evaluated for this analysis based on physical exam and radiographic assessment. Clinically, local control was defined as the absence of physical progression of disease or radiological progression. Physical progression was defined as disease progression requiring any intervention after radiation to protect the airway such as tracheostomy, surgical debulking, or death from upper airway compression. Radiographically, local control was defined as no increase in size greater than 25% posttreatment. At the time of this analysis, two patients had died during radiotherapy with not enough time to assess treatment response. Both of these patients had unresectable disease and received two fractions of radiation before death. These patients were not included in the outcomes measures. Toxicity was measured according to the National Cancer Institute Common Toxicity Criteria for Adverse Events version 4.0. Toxicity was scored weekly using CTC criteria during the radiation on treatment visit. After completion of treatment, patients were followed up approximately every 4 weeks at Vanderbilt University Medical Center.

### 2.4. Statistics

All patients who survived treatment were considered eligible for assessment. Outcomes and survival were assessed for all patients. Continuous features were described using means, medians, and ranges whereas categorical features were summarized with frequency counts and percentages. All data were calculated from the time of pathological diagnosis. All patients were followed up until death or last documented follow-up.

## 3. Results

### 3.1. Patient Characteristics

Seventeen patients were diagnosed with anaplastic thyroid carcinoma and completed treatment with hypofractionated radiotherapy between 2004 and 2012. Nine patients were female and 8 were male (M : F ratio, 1 : 1.1). The median age of diagnosis was 70 years (range, 58–84 years). The median Karnofsky performance status was 70 (range, 50–80). Patient and tumor characteristics are described in Tables 1, 2, and 3. All 17 patients had ATC confirmed by pathological review at our institution. The majority of patients (88%) received concurrent chemotherapy, which consisted of weekly paclitaxel (135 mg/m^2^) or weekly carboplatin (AUC 1-2) and paclitaxel (135 mg/m^2^).

### 3.2. Treatment

Fourteen patients underwent surgical resection prior to radiation (82.3%): ten patients underwent a total thyroidectomy and 4 patients underwent a partial thyroidectomy. Three patients were deemed inoperable at the time of diagnosis: 1 patient due to medical comorbidities and 2 patients due to unresectable disease. The most frequently used fraction size was 3.0 Gy, and the most frequently prescribed dose was 54 Gy in 18 daily fractions (35.3%) with a range of 40–62.5 Gy (Table 3). The median number of fractions was 18 (range, 10–25).

### 3.3. Local Control and Survival

The median survival was 9.3 months. For those with metastatic disease at diagnosis, the median survival was 6.4 months; for those without initial metastases, median survival was 14.2 months. Seven of the 17 patients (41%) were alive one year after initial diagnosis. Two of the 8 patients with metastatic disease at diagnosis were alive after one year, while 5 of the 9 patients (56%) without initial metastases were alive after one year.

Eight patients (47%) had metastatic disease at the time of diagnosis. The median time from diagnosis to metastasis was 2.1 months, and 88% of irradiated patients developed metastatic disease. The lungs were the first site of metastasis in 93% of patients. Fourteen patients had died at the time of analysis. Three patients were lost to follow-up after a mean of 12.5 months. These patients were assumed dead with survival calculated from the last date of follow-up.

Fourteen of the 17 patients (82%) maintained local control of disease at the time of death. Two patients required palliative tracheostomies and one patient required surgical debulking after irradiation. In the patients who experienced local progression, 2 had unresectable disease at diagnosis and 1 received a partial thyroidectomy. Four patients required percutaneous gastrostomy tube placement following radiation. Radiation induced esophagitis was the reason for gastrostomy tube placement in 2 of 4 patients.

### 3.4. Toxicity and Feasibility

Treatment related toxicities were graded using the National Cancer Institute Common Toxicity Criteria for Adverse Events version 4.0. Specifically, dysphagia, esophagitis, and radiation dermatitis were analyzed as documented in the medical record. The incidences of grades 1–4 toxicities are listed in Table 4. All patients experienced grade 1 or 2 toxicities. There were no grade 4 toxicities. However, 7 out of 17 patients (41%) experienced at least one grade 3 toxicity. Moreover, patients with grade 3 toxicity in one category were more likely to have grade 3 toxicity in another category (HR = 1.33). Frequently, this was the result of symptomatic overlap between esophagitis and dysphagia. Patients who were deemed unresectable at diagnosis or underwent a partial resection prior to radiation were more likely to develop grade 3 dysphagia during treatment. This appeared to be related to local tumor progression resulting in mechanical obstruction as opposed to radiation induced esophagitis. Patients who underwent a complete resection prior to the start of radiation experienced the fewest toxicities and treatment delays.

All patients completed their radiation course as prescribed. A treatment delay was defined as an interruption between two radiation fractions greater than 5 days or a total treatment time 10 days greater than expected. Three out of 17 patients experienced treatment delays (18%). All three patients required inpatient admission with a mean hospitalization time of 3.6 days. Reasons for a treatment delay were local progression requiring tracheostomy or poor nutritional intake requiring gastrostomy tube placement.

## 4. Discussion

Given the poor prognosis and rapid progression of this disease, early integration of palliative and supportive care is essential when managing these patients. Knowing when and what type of radiation treatment to deliver can be a clinical challenge and requires a comprehensive understanding of the disease's natural history, sequelae of symptoms, treatment effectiveness, and goals of care. Unfortunately, patients with ATC are confronted with an aggressive disease that affects critical respiratory organs and local control remains a primary determinant of quality of life even in the metastatic setting. Results from the present study appear similar to prior studies with regard to local control and survival (Table 5).

Similar to other reports, our data underline the importance of surgical debulking in the management of ATC. Junor et al. reported that patients who underwent a total or partial thyroidectomy had prolonged survival times compared with patients for whom only a biopsy was feasible [7]. Surgical debulking can prevent distressing symptoms such as airway compression and it is recommended even in the presence of metastasis [16, 17]. Furthermore, reducing the burden of local disease may improve the efficacy of adjuvant therapy. However, surgery alone cannot alter the course of this disease [18]. The combination of surgery and radiotherapy is an independent predictor of reduced cause-specific mortality in patients with ATC [19], though the ideal adjuvant radiation regimen remains unclear.

The low incidence and poor survival rates of patients with ATC limit the ability to conduct large Phase III trials. Most of the available evidence for radiotherapy is derived from single institutional retrospective series. Wang et al. reported on 47 patients with ATC who received radiotherapy as either once or twice daily fractionation escalating up to 66 Gy. Median survival was 5.6 months, but patients receiving higher doses of radiation (45–66 Gy) had significantly longer survival times compared to those receiving doses less than 40 Gy (11.1 versus 3.2 months; *P* < 0.001) [13]. In our series, delivering larger doses in a shorter period of time maintained the concept of dose escalation, yielding an average biologically equivalent dose of 70.2 Gy.

Several common treatment schedules are used in radiation oncology including once daily treatments for ~25–35 days, twice daily treatments for ~15–25 days, and hypofractionated treatments for ~1–20 days. Typically, radiation oncologists attempt to achieve the same total effective dose; therefore, if the number of treatments decreases, the total dose per treatment must increase or the dose is delivered twice daily. One of the major challenges with twice daily treatments is the 6-hour break between fractions. The patient spends most of the day in the clinic or arranging transportation to and from it. The second challenge is increased acute toxicity, which is an important consideration when treating the head and neck region.

Toxicity is divided into acute and late-responding effects depending on whether the tissue is more likely to manifest radiation damage around the time of treatment or in the future. Larger radiation doses per day correspond to greater risk of damage in late-responding tissues (such as the spinal cord) as compared to smaller doses given over a protracted course. However, in patients with limited survival, shorter treatment courses are practical because the patient will not live long enough to face the increased risk of long-term side effects, which classically occur many months to years later.

In the pursuit of better outcomes, several studies have examined the use of twice daily accelerated radiotherapy. De Crevoisier demonstrated treatment effectiveness, but 33% of their patients experienced grade III or IV acute mucositis with a significant amount of chemotherapy induced hematologic toxicity [14]. Dandekar et al. reported that greater than 70% of their patients experienced grade III or IV acute dysphagia and esophagitis and many of these patients discontinued treatment, with less than 10% survival at one year [20].

In our series, toxicity with hypofractionated radiotherapy continued to be an issue with 41% of patients experiencing grade 3 toxicities, but no grade 4 toxicities. The important difference between this regimen and others was that acute toxicities were seen near the end of treatment or after the patient had finished their radiation, thereby limiting the number of treatment related breaks and total treatment time. This allowed patients to continue forward with adjuvant systemic therapy or palliative and supportive care as needed. From the perspective of patient comfort, shorter treatment courses are preferable to longer courses, especially in the setting of limited survival.

Local recurrence in this disease can have devastating consequences and patients have a median survival of 66 days after local progression (McIver et al. anaplastic thyroid carcinoma: a 50-year experience at a single institution, 2001). This study also demonstrated that radiation after complete or near complete resection did not improve local control but did delay the time to local progression (5 versus 3 months). However, radiation or surgery did appear to improve survival over palliative care alone. In previous studies by Tennvall et al. and Werner et al., death from local failure was seen in 36% and 24% of patients, respectively. These studies used twice daily treatment schedules of varying doses and chemotherapy regimens [11, 21]. In our study, death attributed to local failure was seen in 18% of patients, though the patients who developed local failure were inoperable at the time of diagnosis or only received a partial resection or biopsy. Similarly, Foote et al. achieved local control in 30% of patients with variable radiation schedules and dual chemotherapy; however, patients with metastatic disease at diagnosis were excluded from their study [22]. These patients account for a significant portion of patients with ATC and were included in our series. Results from the present study suggest that a hypofractionated regimen is as effective as hyperfractionated regimens and while treatment courses were slightly longer, patients were only treated once per day.

There were multiple limitations in this study including the single institutional retrospective nature, small patient numbers, and variable radiation doses. Most patients received upfront resection possibly because of smaller tumors. Therefore, selection bias may have influenced the overall survival. Despite these issues, this treatment approach is unreported in the literature. Results from the present study suggest that a hypofractionated regimen with concurrent chemotherapy is well tolerated with a favorable toxicity profile and rates of local control when compared with previously used fractionation schemes. While there was no formal assessment of quality of life, given the favorable toxicity as well as the convenience of a shorter, once a day treatment regimen, we believe that this regimen may improve the quality of life in these patients with a generally poor outcome. Based on our results, short course hypofractionated radiation therapy appears to be a viable and safe option in the treatment of ATC and remains a sensible approach given the poor prognosis and symptomatic needs of this patient population. Specifically, this protocol may be useful in patients who have difficulty with a twice daily regimen due to either travel or concerns about their ability to tolerate the acute effects of a twice daily regimen.

## Figures and Tables

**Table 1:** Patient demographics.

Patient	Gender	Age	Date of diagnosis	KPS	Size (cm)	PEG post-XRT	First site of metastasis
1	F	70	1/16/04	80	2.6	No	Pulmonary
2	M	58	6/30/05	80	3.2	No	Pulmonary
3	F	84	4/6/06	60	5.7	No	Pulmonary
4	F	60	4/25/06	70	4.0	Yes	Pulmonary
5	F	77	5/18/06	50	5.2	No	None
6	F	72	9/8/06	60	5.0	No	None
7	F	68	1/2/08	80	3	No	Pulmonary
8	M	78	12/19/07	70	5.9	Yes	Skin and chest
9	F	68	12/7/07	80	4	Yes	Pulmonary
10	M	77	12/15/08	70	5.3	No	Pulmonary
11	M	63	4/21/10	70	16	No	Pulmonary
12	M	80	9/15/10	70	6.0	No	Pulmonary
13	F	76	9/15/10	60	6.0	No	Pulmonary
14	M	62	7/21/11	80	2.5	No	Pulmonary
15	M	74	8/31/11	70	8.0	No	Pulmonary
16	M	70	6/11/12	70	2.5	Yes	Pulmonary
17	F	66	6/21/11	80	2.2	No	Pulmonary

**Table 2:** Tumor characteristics.

Patient	TNM stage	Margin	Surgery	Nodal dissection	Survival (months)
1	T4aN0M1	Negative	Total thyroidectomy	Yes	21.9
2	T4aN0M0	Positive	Total thyroidectomy	Yes	23.6
3	T4aN0M0	N/A	None	No	14.2
4	T4aN1aM1	Gross residual disease	Partial thyroidectomy	No	4.7
5	T4aN1bM0	Gross residual disease	Partial thyroidectomy	Yes	2.3
6	T4bN0M0	Negative	Total thyroidectomy	Yes	24.0
7	T4aN1bM1	Gross residual disease	Partial thyroidectomy	No	9.3
8	T4bN1bM1	Positive	Total thyroidectomy	Yes	5.8
9	T4aN1bM0	Positive	Total thyroidectomy	Yes	10.0
10	T4aN1bM1	Positive	Total thyroidectomy	Yes	6.5
11	T4aN1bM0	Positive	Partial thyroidectomy	Yes	23.3
12	T4aN1aM1	N/A	None	No	6.2
13	T4aN0M0	Negative	Total thyroidectomy	No	17.8∗
14	T4aN1bM0	Positive	Total thyroidectomy	Yes	4.4
15	T4bN0M0	Positive	Total thyroidectomy	Yes	7.9
16	T4aN1aM1	N/A	None	No	3.8∗
17	T4aN0M1	Negative	Total thyroidectomy	Yes	16.1∗

^*^Date of last follow-up.

**Table 3:** Treatment characteristics.

Patient	Elapsed days	Completed treatment	Dose	Fraction	Concurrent chemotherapy	Local control
1	29	Yes with delay	5100	17	Yes	Yes
2	23	Yes	5100	17	Yes	Yes
3	37	Yes	6250	25	No	No
4	36	Yes with delay	5700	19	Yes	No
5	27	Yes with delay	5700	19	Yes	Yes
6	29	Yes	5500	22	Yes	Yes
7	23	Yes	5400	18	Yes	Yes
8	24	Yes	5400	18	Yes	Yes
9	24	Yes	5400	18	Yes	Yes
10	13	Yes	4000	10	No	Yes
11	23	Yes	4950	18	Yes	Yes
12	23	Yes	5400	18	Yes	Yes
13	36	Yes	6250	25	Yes	Yes
14	26	Yes	5400	18	Yes	Yes
15	22	Yes	5100	17	Yes	Yes
16	25	Yes	5400	18	Yes	No
17	21	Yes	4500	15	Yes	Yes

**Table 4:** Toxicity.

Toxicity/number of patients (%)				
Grade	Dysphagia	Esophagitis	Dermatitis	Total (*N*)
1	7 (45)	9 (53)	7 (45)	17 (100)
2	6 (35)	5 (29)	6 (35)	17 (100)
3	4 (24)	3 (18)	4 (24)	7 (41)
4	0	0	0	0

**Table 5:** Major radiation series comparing outcomes of anaplastic thyroid carcinoma.

Author	Intent	Median radiation dose	Resection	Fractionation	Local control	Chemotherapy	Radiation duration	Median survival
Wang et al. [13]	Definitive	60 Gy (45–66)	61%	Once daily or bid	94% (6 months)	No	4–6 weeks	11.1 months
	Palliative	40 Gy (split course)	50%	Once daily	65% (6 months)	16% (prior to or during)	4–6 weeks	3.2 months

de Crevoisier et al. [14]	Definitive	40 Gy (later boost to 50–55 Gy)	71%	Bid	68% (45 median FU)	Prior to and after	3-4 weeks	10 months (both arms)
	Palliative	40 Gy (later boost to 50–55 Gy)	50%	Bid	68% (45 median FU)	Prior to and after	3-4 weeks	

Dumke et al. [15]	Both	50 Gy (6–60.4)	80%	Daily or bid	Unknown	15%	Unknown	5 months; if >50 Gy 10.5 months

Stavas et al.	Definitive	54 Gy (49.5–62.5)	89%	Daily	89% (at time of death)	88% (concurrent)	23.5 days (13–36)	14.2 months
	Palliative	54 Gy (40–57)	75%	Daily	75% (at time of death)		26 days (22–37)	6.4 months
